# Angler perceptions of pelican entanglement reveal opportunities for seabird conservation on fishing piers in Tampa Bay

**DOI:** 10.1371/journal.pone.0320424

**Published:** 2025-03-25

**Authors:** B. Alexander Simmons

**Affiliations:** Tampa Bay Estuary Program, St. Petersburg, Florida, United States of America; Tsinghua University, CHINA

## Abstract

Injuries from entanglement in marine debris are a significant threat to seabirds globally, and fishing gear is the most common debris affecting seabirds. In Tampa Bay, Florida, entanglement of brown pelicans (*Pelecanus occidentalis*) at fishing piers has been a highly contentious issue for years, especially at the Skyway Fishing Pier State Park. With entanglements continuing to rise, new fishing regulations were adopted at the pier to reduce the likelihood of severe entanglements, which include a controversial seasonal ban on certain high-risk fishing gear during the pelican breeding season. To better understand the extent of this problem and identify potential leverage points for promoting behavioral change, this study analyzed data on pelican condition and abundance near the south Skyway Fishing Pier, as well as data from questionnaires conducted on the pier capturing anglers’ perceptions of the issue and several proposed solutions, including the new gear restrictions. The results suggest a potential attraction of pelicans to anglers, with entanglement risks greatest at peak locations and times of angler activity. However, results from the angler surveys highlight several opportunities for minimizing these risks. Overall, anglers are generally concerned about the issue, believe others are also concerned, and supportive of educational videos and greater enforcement or punishment for people feeding pelicans. Anglers had mixed opinions on the new gear restrictions. Five types of anglers were identified using audience segmentation techniques, with each type representing a different potential target audience for promoting behavior change on the pier. Depending upon the audience, strategic messages using frames that focus on leadership or self-identity may be most effective for increasing the number of anglers who can safely rescue a hooked bird. Recommendations for future analyses and pilot social marketing campaigns are discussed to support further investigation into the human dimensions of seabird conservation in Tampa Bay.

## Introduction

Marine debris presents a substantial threat to marine wildlife globally [1], and seabirds are one of the most vulnerable to entanglement and ingestion of these lost or discarded materials [1–3]. Entanglements in marine debris have been documented in more than one-third of the world’s seabird species, with fishing gear (e.g., monofilament lines, hooks) being the most common type of debris [4]. Several studies have found that incidences of seabird entanglement are among the highest in pelican species [4–6], which may be due to their plunging-style of foraging and the risk of fishing line being entangled in the dense vegetation at their roosting sites [1]. Brown pelicans (*Pelecanus occidentalis*), in particular, appear to have a high rate of entanglement throughout the United States [5]. For example, brown pelicans were among the seabirds most affected by entanglements in California [7], and in the Monterey Bay region, rescued brown pelicans were four times as likely to suffer from fishing gear injuries than any other type of debris [8].

The sources of these entanglements are often unknown [1]. To date, most research has focused on the impacts of commercial or industrial fisheries on marine wildlife entanglement, with few studies explicitly investigating the impacts from recreational fisheries [9–11]. However, the recreational fisheries industry continues to grow rapidly, particularly in high-income nations like the United States [12], and case studies are just starting to emerge that highlight recreational anglers’ contributions to seabird entanglement [13,14]. In response, a variety of interventions have been proposed or adopted around the world to reduce the impacts of fishing gear on seabirds and other marine wildlife. These can range from international policies regulating industrial fishing practices and the use of high-risk materials, to localized interventions targeting individual angler behaviors [4,10]. While top-down interventions, such as command-and-control regulations, are common within recreational fisheries, practitioners are frequently searching for alternative, bottom-up solutions to reducing the frequency of seabird entanglement. For example, the Queensland Government in Australia launched a Fishing Line Recovery Bin program to install more bins for discarding used fishing gear at key fishing locations, supplemented with educational signs communicating the importance of proper gear disposal for the environment [6]. The “Seal the Loop” campaign, created by Zoos Victoria (Australia), similarly aimed to reduce entanglements by raising awareness about the issue, promoting recycling messages, and installing more gear disposal bins [15]. These alternative approaches emphasize the voluntary nature of angler behavioral change and the role that the social sciences could play in reducing wildlife entanglements.

### A contentious issue in Tampa Bay

Tampa Bay is an important destination for migratory brown pelicans, especially during the winter breeding season [16]. Pelicans, as well as other seabird species, are frequent visitors to the fishing piers located throughout the watershed, which has ultimately led to frequent incidents of entanglement, injury, and death from fishing gear [17]. Although published data on these entanglements are scarce, Thomas and Forys [14] documented and compared the rates of brown pelican entanglement across four fishing piers in Tampa Bay during 2019-2020. The authors found that 7% of all pelicans counted were entangled by fishing gear; entanglements were most frequently observed at the Skyway Fishing Pier State Park’s south pier, the longest fishing pier in the world [18]. In an effort to reduce the number of pelican deaths from fishing gear, the Florida Fish and Wildlife Conservation Commission (FWC) launched the “Don’t cut the line” educational campaign in 2016, with a goal of educating anglers on the proper steps to rescue a hooked bird [19]. However, severe entanglements continued to occur [17]. In November 2022, FWC announced they would be holding public hearings to discuss potential solutions to the entanglement problem [20]. These hearings were frequently fraught with debate between angler and pelican interest groups, with each side placing blame on the other for the continued rise of entanglements [21].

In July 2023, FWC announced its decision to adopt new regulations at the north and south piers of the Skyway Fishing Pier State Park [22]. As of 1 October 2023, the following changes are now in effect: (1) anglers must have proof of completing an annual education requirement (an online education and training course on entanglement at fishing piers); (2) anglers are limited to the use of no more than two sets of hook-and-line fishing gear (previously allowed three sets); and (3) anglers are prohibited from using a fishing rig with more than one hook attached, as well as using any multiple hook, from 15 November to 15 March every year (i.e., during the pelican breeding season). In their announcement, FWC stated that the purpose of the regulations was to “reduce the likelihood and severity of seabird entanglement,” and that “FWC will monitor the effectiveness of these regulations [...] two years after implementation” [22]. Public response to the announcement was mixed, with anglers largely unsupportive of the gear restrictions and pelican advocates arguing that the regulations did not go far enough [23]. However, the effectiveness of such gear bans have yet to be reliably quantified, and at the time of the announcement of the Skyway Pier regulations, FWC did not have a plan prepared for estimating the effectiveness of these new regulations after two years.

### The need for behavioral change

State agencies have now implemented a wide variety of tactics to try to reduce pelican deaths from entanglement at the Skyway Pier, including regulations, gear disposal bins, educational campaigns, and even on-site rescuers. For complex social-ecological issues like wildlife entanglement in fishing gear, the optimal solution is likely some combination of top-down and bottom-up intervention. However, identifying the optimal strategy for changing angler behavior will require more research into the human dimensions of pelican entanglement. Indeed, other studies have gained important insights into the factors influencing anglers’ use and disposal of high-risk gear by applying psychological theories and social marketing principles [24,25]. A crucial component of any behavior change intervention—including regulatory intervention—is messaging. Traditional conservation outreach tactics, such as FWC’s “Don’t cut the line” campaign, often rely on the deficit model of behavior [26], which assumes that knowledge or awareness is enough to change behaviors [27,28]. However, countless studies have shown that education is not enough; behaviors are influenced by a variety of demographic, socioeconomic, and psychosocial factors, especially conservation-related behaviors [29]. ‘Strategic messages’ are explicitly designed to influence the response of target audiences by harnessing the influence of these behavioral drivers, such as values, identity, and social norms [30,31]. While this approach is common in fields like public health [32], it remains underutilized in conservation [26] and may be an important complement to top-down intervention in the contentious case of the Skyway Fishing Pier.

The purpose of this study is to provide a baseline understanding of the human dimensions of pelican entanglement at the South Skyway Fishing Pier by combining social and ecological data to present a more complete picture of the extent of the problem and identify potential leverage points for promoting behavioral change. First, pelican and angler observations are collected to estimate the prevalence of pelican entanglements on the pier and identify important site-specific factors contributing to entanglement risk. Second, social data is collected directly from anglers on the pier to explore differences in perceptions of pelican entanglement and potential solutions, including the new gear restrictions established by FWC. Finally, audience segmentation is conducted to identify different types of anglers on the pier and illuminate opportunities for using strategic messages to promote behavioral change in each target audience. By better understanding entanglement risks and important differences within the angler population, future behavior change campaigns will be able to test the effects of tailored messages on different types of anglers, which may be the crucial missing ingredient for reducing seabird entanglements in Tampa Bay.

## Methodology

### Study area

**Fig 1:**
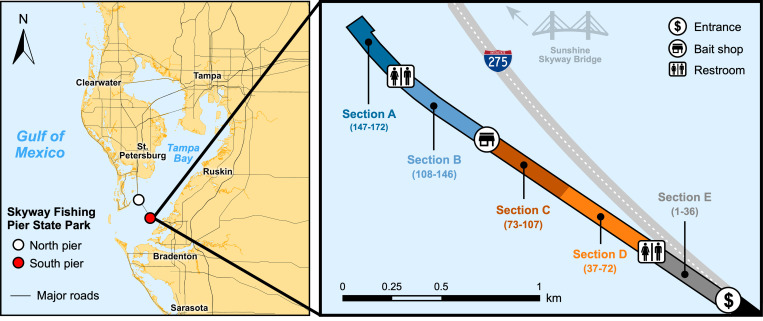
Study area. Map of the five sections (and marker numbers) of the south pier of Skyway Fishing Pier State Park in Tampa Bay, including points of interest used to delineate sections. The scale of the pier has been exaggerated from original satellite images for easier viewing and is therefore for illustrative purposes only.

The south pier of the Skyway Fishing Pier State Park is located south of the Sunshine Skyway Bridge in Manatee County, Florida, accessible off Interstate 275 (Fig 1). Part of the network of Florida State Parks, the Skyway Fishing Pier is open 24 hours a day and requires paid entry, which provides visitors with unlimited access to both the north and south piers in a given day. The south pier is the longest, spanning nearly 2.5 km (compared to the 1 km-long north pier), with parking allowed across most of the pier’s length. Markers are painted on the pier from 1 (entrance) to 172 (end of pier) in approx. 14-15 m intervals. For this study, five sections of the pier were delineated using these markers (Sections A, B, C, D, E), as well as notable points of reference when possible, such as restrooms and the on-site bait shop. In many spots, remnants of the old, decommissioned fishing pier remain standing, which can be 6-15 m from the current pier depending upon the location. Visitors cannot access the old pier, but it is a popular spot for seabirds to perch.

### Pelican surveys

Pelican surveys were conducted on the South Skyway Fishing Pier over 68 days between 22 September 2023 and 30 April 2024. Surveys were conducted by the Tampa Bay Estuary Program prior to the implementation of the new gear restrictions (22 September to 11 November), after which surveys were continued under FWC (22 December to 30 April). Surveys were originally planned to occur twice per week on an alternating schedule of Wednesdays, Fridays, Saturdays, and Sundays to capture days with high and low numbers of visitors to the pier. Tuesdays were added to the sampling schedule under FWC. Prior research by Thomas and Forys [14] showed that the frequency of injured and dead pelicans near the fishing pier was significantly higher during the morning (7am-11am) and mid-day (11am-3pm). Therefore, surveys were only conducted during the same morning and mid-day periods on an alternating schedule. Each survey started at marker 37 (Section D) and ended at marker 172 (Section A). Section E was excluded from the survey transect due to several unique confounding factors that would reduce the reliability of comparisons with other sections, including the high volume of traffic near the park entrance, greater presence of park staff and law enforcement, limited parking spaces, and noise from the adjacent highway (I-275). A total of 69 surveys were conducted by the end of the sampling period.

Field researchers collected the following pelican data at each pier marker location: the number and location of adult and juvenile brown pelicans, number of injured adult and juvenile pelicans at different levels of injury severity, and number of dead adult and juvenile pelicans. Pelicans were only counted if they were located within one of the following areas: directly on the pier, on the remnants of the adjacent old pier, or in the water within a distance equivalent to the distance of the old pier. Pelicans that were spotted farther from the pier or flew by without landing near the pier were not counted. Therefore, estimates of pelican abundance only reflect the number of pelicans in close proximity to the pier, and thus those that are at greatest risk from entanglement. Field researchers were trained to identify differences between adult and juvenile pelicans, as well as identifying and classifying injuries from fishing lines or hooks. Injuries were classified into minor, moderate, and severe levels. ‘Minor’ injuries occur when a hook is present without an attached fishing line that doesn’t appear to affect mobility for flight or foraging. ‘Moderate’ injuries consist of hooks with a trailing fishing line, as well as minor swelling or wounds, that don’t appear to be affecting mobility but have the potential to become a severe injury or entanglement. ‘Severe’ injuries represent any condition in which fishing line is wrapped around a body part and/or severe swelling or wounds are present that may substantially reduce mobility. Two measures of angler pressure were also collected during the pelican surveys at each pier marker: the number of active anglers and the number of fishing lines in the water. ‘Active’ anglers are defined as anglers that were actively engaged in fishing at the time of recording; anglers who were in their vehicle, loading/unloading equipment, or otherwise not monitoring a fishing line were not counted. Fishing lines that were not in the water, such as those that were on the ground or in the back of vehicles, were considered not in use and not counted during the surveys. For all analyses, the number of lines per angler was calculated and used as a replacement for the total number of fishing lines as an indicator of the relationship between anglers and fishing lines, due to the exceptionally high correlation between the total number of anglers and fishing lines (Pearson’s *r* =  0.986, *p* <  0.001). Given the observational nature of this research, no permit was required, provided that park entry fees were paid [33]. A permit was sought from the Florida Department of Environmental Protection to waive park entry fees on 7 August 2023 and was declined on 17 November 2023. All data collected from 22 September to 11 November 2023 complied with the state’s requirement for payment of normal entry fees.

### Abundance models

Three models were constructed to identify factors predicting the abundance of all pelicans, injured pelicans, and anglers at the pier. To reduce the frequency of zeroes, counts at each marker were aggregated to their respective sections, such that the unit of analysis was the total number of sightings in a given section during a given survey (i.e., four observations per survey). For the models, data collected during Tuesdays and Thursdays were excluded from analysis since they were not equally surveyed in the restricted and unrestricted seasons. Given the frequency of zero pelican sightings in some sections of the pier, zero-inflated Poisson regressions [34] were used to model total and injured pelican abundance using the ‘pscl’ package in R [35]. Coefficient estimates are presented as odds ratios (OR) with accompanying 95% confidence intervals (CI). A linear regression model was used to identify predictors of angler abundance, using diagnostic plots of the residuals (i.e., linearity, normality, homoscedasticity, and outliers/leverage points) to evaluate any potential violations in model assumptions. Angler abundance was strongly right-skewed, resulting in poor model diagnostic tests. A square root transformation was applied to angler abundance, which improved diagnostics, with the exception of strong outliers from a survey conducted on Saturday, 10 February 2024, where only three anglers were recorded on the entire pier. After removing the outliers, diagnostic tests indicated no remaining issues. Coefficient estimates for this model are thus based on the square root of angler abundance for slightly fewer surveys than the pelican abundance models.

### Angler surveys

Anglers on the South Sunshine Skyway Fishing Pier were randomly recruited for participation in this study. In-person surveys were administered on the pier twice per week (16 days) between 22 September and 11 November 2023, at the same time as pelican surveys. Each day, the start point was randomly selected between Sections A and C of the pier, and all anglers present down the length of the pier from the start point were offered the opportunity to complete the questionnaire until the pelican surveys were completed (approx. 2 hours). Participants were allowed to complete the survey on paper or answer questions verbally, depending upon their preference. Two versions of the survey were designed: a full survey and a condensed version. Anglers who participated in the full survey were offered an incentive in the form of a free tackle box of 100 circle hooks. The desired sample size for the full survey was 100 anglers, and this sample was reached after 13 days. After running out of tackle boxes, 57 anglers were recruited to take a condensed version of the survey for the last three days, which only consisted of questions about their pelican observations on the pier (see *Pelican observations*). Surveys were available in English, Spanish, and Vietnamese, though no participants completed the Vietnamese version. Although participants were allowed to repeat the survey multiple times, no participants completed the survey more than once, and thus no issues of pseudoreplication are present. The study protocol was approved by the U.S. Environmental Protection Agency (EPA) (Hooked Birds Campaign Phase I: Monitoring Entanglement of Brown Pelicans [*Pelecanus occidentalis*] at the South Skyway Fishing Pier Quality Assurance Project Plan) prior to data collection under the EPA’s Generic Clearance for Citizen Science and Crowdsourcing Projects (ICR No. 2521.17) and adheres to the American Association for Public Opinion Research’s Code of Professional Ethics and Practices [36] and the Tampa Bay Estuary Program’s Quality Management Plan [37]. Informed consent (written or verbal, depending upon their preference) was obtained from all participants.

### Demographic and trip characteristics

A copy of the survey is available in S1 File. The survey included questions to capture the following demographic characteristics: age, gender, employment status, income, ZIP code, and Florida resident status. Those who were out-of-state residents were presented with additional questions regarding their reasoning for visiting Florida and the US state or country they came from. Several questions also asked about their trip to the pier that day. Participants were asked what time of day they had been on the pier, with options for morning (7am-11am), mid-day (11am-3pm), evening (3pm-7pm), and late night or early morning (7pm-7am). This question was used to determine which anglers had been on the pier outside of the period in which pelican surveys were conducted. Participants were also asked their reason for going to the pier (e.g., fishing for fun/sport, fishing for their own food or food to sell, watching friends/family fish), who, if anyone, they went to the pier with that day (e.g., friend, parent, spouse/significant other), and how often they go to the pier in a typical year (once a week, once a month, a few times per year, or first time at the pier). Participants were also provided a map of the pier sections and asked in which section they spent their time. However, anglers frequently struggled to identify the section they were in, so after the survey was completed, their answer was verified and amended, if needed. For analyses, the following variables were converted into binary indicators to reduce model complexity: gender (1 =  male, 0 =  female), employment status (1 =  full-time, 0 =  other status), reason for pier visit (1 =  fishing for own food *or* food to sell, 0 =  other reasons), and company on the pier (1 =  fishing with others, 0 =  fishing alone). Frequency of pier visits was also recategorized into the following: ‘rarely’ =  first time at the pier *or* a few times per year, ‘occasionally’ =  once or twice a month, and ‘frequently’ =  at least once a week.

### Fishing gear

Although the fishing gear restrictions had not begun during the time of surveying, it is possible that anglers might still be reluctant to explicitly say if they were using gear that would eventually be banned during the pelican breeding season (i.e., multi-hook rigs and treble hooks). To estimate the prevalence of these two sensitive types of gear among anglers, two questions were designed using the unmatched count technique [38]. Participants were randomly assigned to receive a ‘control’ or ‘treatment’ version of the survey. In the control group, one question presented participants with a list of four types of tackle (monofilament line, swivel, braided line, sinker or weight), and participants were asked how many of these different types of tackle they used while they were at the pier that day (‘none of them,’ ‘one of them,’ ‘two of them,’ etc.). A second question was designed in the same way but focused on four types of terminal tackle (bobber, circle hook, artificial bait, natural bait). Participants in the treatment group received the same two questions, but with the addition of the sensitive gear items—a multi-hook rig to the list of tackle and a treble hook to the list of terminal tackle—and response options were updated to reference five types of gear instead of four. To estimate the prevalence of multi-hook rigs and treble hooks, response options were converted into their numerical equivalents (0 to 5), and the estimated proportion of anglers using these sensitive types of gear was calculated as the difference in the mean number of items selected between the treatment and control groups [38,39]. Mann-Whitney tests [40,41] were performed to confirm differences between treatment and control groups. Linear regression models were also used to identify potential influences of age, gender, income, and fishing for food on the number of gear cited in the treatment and control groups.

### Pelican observations

Participants were asked if they had seen any pelicans on or near the pier during their trip. Those who answered ‘yes’, were asked to estimate the number of pelicans they saw. These participants were also asked if (and how many) pelicans they saw that appeared to be dead or injured/entangled by fishing lines. An additional question asked if they saw anyone feeding pelicans on or near the pier, and if so, what was being fed to them (dead fish or used bait, chum, human food, something else, or not sure). Participants who gave inconsistent answers, such as saying no pelicans were seen but still entering a number, or vice-versa, were excluded from subsequent analyses of this data. One participant who stated they saw 100 pelicans was also excluded as an extreme outlier. These eight questions composed the condensed version of the survey that was used after all tackle box incentives were given away. For comparison with objective pelican survey data, pelican observations were classified into one of the following bins to permit some degree of error in counting or recall: 0, 1-4, 5-9, 10-19, and 20 + . Cut-off values of 1, 5, 10, and 20 were chosen given typical cognitive biases in number preference, or ‘heaping’ [42]. Anglers whose subjective estimates were within the same bin as the objective estimates for their respective section of the pier were considered ‘about right’ in their estimation. Anglers whose estimates were one or more bins below the objective estimate’s bin were considered an ‘underestimate,’ and those who were one or more bins above were considered an ‘overestimate.’ Anglers whose time on the pier extended beyond the period in which objective pelican data was collected were not evaluated on their accuracy. Fisher’s exact test [43] was performed to determine if the number of more- or less-accurate anglers differed significantly between sections of the pier.

### Psychosocial characteristics

Four items measured anglers’ perceptions of pelican entanglement. Participants were asked to rate their level of agreement with statements regarding their concern about pelicans dying from entanglement, other anglers’ concern about pelicans dying, their confidence in their ability to safely untangle a hooked pelican, and their knowledge of best practice when saving a hooked pelican (Table 1). Participants were also presented with five actions that some stakeholders have proposed as potential solutions to the pelican entanglement problem and asked to rate how effective they thought each one would be at reducing pelican deaths from entanglement, including gear restrictions, “no fishing” zones, demolishing the old pier, creating online training videos, and greater enforcement or punishment for feeding pelicans. One question asked participants if they were aware of the upcoming gear restrictions on the South Skyway Fishing Pier. Participants who answered “yes” were permitted to answer the remaining questions about the gear restrictions. Many participants who answered “no” asked for an explanation of the new restrictions. For these participants, the restrictions were explained to them, and they were allowed to answer the remaining questions using this newfound knowledge. Participants who indicated they were unaware of the upcoming restrictions, and did not enquire about them, were excluded from analysis of the remaining questions. These remaining questions consisted of eight statements regarding their perceptions of the upcoming gear restrictions, such as if the restrictions would affect them, if they would be difficult to follow, and their anticipation of descriptive and subjective compliance norms.

**Table 1:** Survey items measuring the psychosocial characteristics of anglers.

Survey item	Scale
*Perceptions of entanglement*	
I am concerned about the number of pelicans dying from fishing lines and hooks	1. Strongly disagree 2. Disagree 3. Somewhat disagree 4. Somewhat agree 5. Agree 6. Strongly agree
Most anglers are concerned about the number of pelicans dying from fishing lines and hooks	
The best way to save a pelican caught in a fishing line is to cut the line as fast as possible^a^	
If a pelican was entangled in my fishing line, I am confident that I could safely untangle it	
*Perceptions of proposed solutions*	
Limiting the gear that anglers can use on the pier	1. Not effective at all 2. Slightly effective 3. Moderately effective 4. Very effective 5. Extremely effective
Demolishing the remains of the old pier where many birds perch	
Having a “no fishing zone” in parts of the pier with more pelicans	
Create online videos showing how to safely untangle pelicans from fishing line	
Greater enforcement or punishment for people caught feeding pelicans on or near the pier	
*Perceptions of upcoming gear restrictions*	
The new restrictions will not affect me^a^	1. Strongly disagree 2. Disagree 3. Somewhat disagree 4. Somewhat agree 5. Agree 6. Strongly agree
It will be difficult for me to follow the new restrictions	
The new restrictions do not go far enough to prevent pelican deaths on the pier^a^	
The new restrictions do not address the main cause of pelican deaths^a^	
There will be very little enforcement of the new restrictions^a^	
Most anglers will follow the new restrictions	
The people who are important to me will care if I follow the new restrictions	
The new restrictions will probably reduce the number of pelican deaths from fishing lines	

^a^Reverse coded for analysis.

### Audience segmentation

A hierarchical cluster analysis was performed to identify unique angler typologies based upon the four items measuring anglers’ perceptions of entanglement: personal concern, descriptive norms of concern, knowledge of best disentanglement practice, and confidence in disentanglement ability. To avoid cluster algorithm dependence on arbitrary variable units, values for the four items were rescaled to z-scores. Agglomerative coefficients (*ac*) were compared between multiple cluster methods, with Ward’s method for hierarchical agglomerative clustering using squared Euclidean distance [44] producing the strongest clustering structure (*ac* =  0.949). Ward’s clustering solutions with up to 10 clusters were compared across several measures of structural fit, including the elbow method [45], average silhouette width [46], and gap statistic [47]. Collectively, these measures suggested an optimal cut-off point between five to seven clusters. Given the small sample size, the five-cluster solution was selected to ensure each cluster had at least 10 anglers assigned to it. Within each cluster, variables with a mean z-score greater than the 95% confidence interval of the group mean (95% CI =  ± 0.199) were considered ‘high,’ and variables below the CI were considered ‘low.’ Variables with means beyond 1 standard deviation (SD) of the mean were considered ‘very high/low.’ Variables within the CI were not considered a defining characteristic of the cluster.

Kruskal-Wallis H tests [48] were first performed to identify differences between clusters across numerical variables, including age, perceptions of proposed solutions, and perceptions of upcoming gear restrictions. A post hoc Dunn’s test with Bonferroni correction [49] was performed on variables identified by the Kruskal-Wallis tests to identify significant differences in pairwise cluster comparisons. For categorical variables, including demographics, trip characteristics, and pelican estimate accuracy, Fisher’s exact test was used to identify significant differences between clusters. A series of logistic regression models were then constructed for each cluster to identify predictors of cluster membership from the variables identified by the Kruskal-Wallis tests and Fisher’s exact tests. Each model underwent sequential parameter reduction until the most parsimonious model explaining cluster membership was achieved and improvements to the Akaike information criterion (AIC) began to diminish. McFadden’s pseudo *R*^*2*^ [50] was used to assess model fit. All analyses were performed in R version 4.3.3 [51].

## Results

### Pelican abundance

A total of 2,737 pelicans were recorded on or near the pier over the 69 surveys, the majority of which were juveniles (77%). On average, more pelicans were counted during the gear restriction season (*mean ±  SD*; 45 ±  25 pelicans per survey) than during the unrestricted season (34 ±  36 pelicans per survey). Particularly during the months preceding the restriction season (September to November), it was not uncommon to see few or no pelicans within close proximity of the pier. Overall, more pelicans tended to be near the pier during mid-day (45 ±  34 pelicans) compared to mornings (34 ±  28 pelicans per survey). Section A of the pier had a notably higher average abundance of pelicans (16 ±  15 pelicans per survey) than other sections, which ranged from 7 ±  9 (Section C) to 9 ±  11 pelicans per survey (Section B), though there was exceptionally high variability. A total of 150 injured pelicans (5.48% of all pelicans) were recorded during this period, 81% of which were juveniles. Injuries were more frequently observed during the gear restriction season, in which 109 pelicans had noticeable injuries. This represents 6.92% of all pelicans counted during this season. In contrast, only 41 injured pelicans were counted during the unrestricted season (3.53% of all pelicans counted in this season). During the unrestricted season, the frequency of minor, moderate, and severe injuries were relatively equal (32-34% of injured pelicans). During the restriction season, severe injuries were less common (22%) but moderate injuries were much more common (47%). Only two dead pelicans (both juveniles) were recorded during the survey period. See S1 Table for comparisons of pelican abundance across all survey days, times, and locations.

Under the zero-inflated models, both injured and uninjured pelicans are significantly more likely to be absent near the pier during the unrestricted season (Table 2). Sections B and C are also more likely to have no pelicans near the pier than sections A and D. When pelicans are present (count model), their abundance is significantly greater during the restriction season, during mid-day, and in sections A and B of the pier. After controlling for these confounding factors, areas with greater numbers of anglers are also significantly more likely to have more pelicans nearby, though the influence is relatively small (OR =  1.004, 95% CI =  [1.001, 1.007], *p* =  0.015). When injured pelicans are present, they are also more abundant in sections A and B of the pier. However, no other factors predicted the abundance of injured pelicans.

**Table 2:** Predictors of pelican abundance near the pier, for all pelicans and injured pelicans.

Variable	Pelican abundance		Injured pelican abundance	
	Zero-inflated model	Count model	Zero-inflated model	Count model
	OR [95% CI]	OR [95% CI]	OR [95% CI]	OR [95% CI]
(Intercept)	0.77 [0.17, 3.50]	6.07 [4.85, 7.59]^a^	12.08 [0.78, 187]	1.18 [0.31, 4.52]
Season				
Restrictions	0.21 [0.10, 0.48]^a^	1.24 [1.12, 1.38]^a^	0.07 [0.01, 0.32]^a^	0.93 [0.44, 2.01]
Time of day				
Mid-day	0.67 [0.34, 1.34]	1.35 [1.24, 1.48]^a^	0.27 [0.07, 1.04]	0.8 [0.47, 1.37]
Pier section				
C	5.44 [1.87, 15.79]^a^	1.11 [0.96, 1.28]	5.05 [0.53, 48.36]	1.82 [0.74, 4.44]
B	3.88 [1.26, 11.96]^a^	1.31 [1.14, 1.50]^a^	7.45 [0.77, 71.91]	3.46 [1.56, 7.65]^a^
A	2.49 [0.77, 8.00]	2.10 [1.85, 2.38]^a^	2.73 [0.29, 25.53]	2.47 [1.11, 5.53]^a^
Number of anglers	0.99 [0.97, 1.02]	1.004 [1.001, 1.007]^a^	0.98 [0.94, 1.01]	0.99 [0.97, 1.01]
Lines per angler	0.43 [0.16, 1.14]	1.02 [0.88, 1.18]	0.41 [0.08, 2.12]	0.66 [0.30, 1.44]
Log likelihood	-1123		-190.4	

Estimates represented as odds ratios (OR) and the 95% confidence interval (CI).

^a^Significant at α = 0.05.

### Angler pressure

A total of 4,669 anglers were recorded on the pier during the survey period, with an average of 43 ±  30 anglers per survey (*mean ±  SD*) during the gear restriction season and 93 ±  62 anglers per survey during the unrestricted season. Overall, more anglers tended to be present during mid-day (77 ±  55 anglers per survey) than mornings (58 ±  53 anglers per survey) and in sections A (23 ±  17 anglers per survey) and B (22 ±  18 anglers per survey) than in sections C (13 ±  12 anglers per survey) and D (10 ±  12 anglers per survey). Sundays were generally the most popular day for anglers (116 ±  78 anglers per survey) and Wednesdays were the least popular (36 ±  23 anglers per survey). Although it was not uncommon to spot some unattended fishing lines on a given day, there was an exceptionally high correlation between the number of anglers and the number of fishing lines in the water in each section of the pier (Pearson’s *r* =  0.986, *p* <  0.001). On average, there were 1.29 lines counted per angler during both seasons, with slightly more counted in section B (1.33 ±  0.45 lines per angler) than in section C of the pier (1.16 ±  0.44 lines per angler). See S2 Table for comparisons of angler pressure variables across all survey days, times, and locations. Nearly all of these differences were found to be significant predictors of angler abundance on the pier (Table 3). Overall, angler pressure is higher during the unrestricted season, during mid-day, on weekends, and increases further toward the end of the pier.

**Table 3:** Predictors of angler abundance on the pier.

Variable	Coefficient (SE)^a^	p-value
(Intercept)	1.97 (0.24)	< 0.001
Season		
Restrictions	-1.34 (0.16)	< 0.001
Time of day		
Mid-day	0.65 (0.16)	< 0.001
Day of week		
Friday	0.57 (0.22)	0.010
Saturday	2.20 (0.23)	< 0.001
Sunday	2.30 (0.22)	< 0.001
Pier section		
C	0.60 (0.22)	0.008
B	1.74 (0.22)	< 0.001
A	1.83 (0.22)	< 0.001
Adjusted *R*^*2*^	0.624	

^a^Coefficients are based on the square root transformation of angler abundance.

### Angler characteristics

A total of 100 anglers participated in this study. Participants were predominantly male, Florida residents, and employed full-time (Table 4). Millennials were the most common generational cohort (39%), followed by Baby Boomers and older generations (23%), with a variety of income categories represented. Only 6% of participants were visiting the pier for the first time; otherwise, there was a relatively similar number of participants who visited the pier more or less frequently. The majority of participants stated that they were fishing for fun or sport (86%), with fishing for their own food (40%) being the second most popular reason for visiting the pier. One-third of participants came to the pier alone; those that were fishing with others were often with a spouse/significant other (27%) or a friend (22%). Half of the participants were surveyed in Section B of the pier, and the others were nearly equally spread between Sections A and C.

**Table 4:** Descriptive characteristics of participating anglers.

Demographic characteristics		Trip characteristics	
Variable	Frequency	Variable	Frequency
Residency		Frequency of visits^a^	
Florida resident	91%	First time at the pier	6%
Out-of-state resident	9%	A few times per year	30%
Gender^a^		Once or twice a month	35%
Male	84%	At least once a week	28%
Female	15%	Reason for visit^b^	
Age^a^		Fishing for fun or sport	86%
Generation Z	16%	Fishing for own food	40%
Millennial	39%	Fishing for food to sell	1%
Generation X	18%	Watching friends/family fish	13%
Baby Boomer (or older)	23%	Something else	5%
Employment		Company on pier^b^	
Full-time	69%	Alone	33%
Part-time	7%	Friend	22%
Student	3%	Parent/sibling	10%
Retired	17%	Spouse/significant other	27%
Unemployed	4%	Children	15%
Income^a^		Someone else	1%
$25,000 or less	12%	Location on pier	
$25,001 - $50,000	21%	Section A	26%
$50,001 - $75,000	33%	Section B	50%
$75,001 - $100,000	16%	Section C	24%
$100,000 or more	11%	Section D	0%

^a^Not answered by all anglers.

^b^Multiple answers allowed.

### Fishing gear

One angler skipped the fishing gear question, leaving 49 anglers in the control group and 50 anglers in the treatment group. On average, anglers in the control group cited using 2.63 ±  0.18 (*mean* ±  *SE*) types of tackle, while 3.38 ±  0.19 types of tackle were cited by those in the treatment group. The difference in group means (0.75 ±  0.26), suggests that 75% of anglers are likely using multi-hook rigs on the pier during this period. The difference between groups was statistically significant (Mann-Whitney test, W =  852, *p* =  0.008). For terminal tackle, an average of 2.78 ±  0.14 and 2.80 ±  0.17 types of gear were cited by the control and treatment groups, respectively. This led to an insignificant difference between groups (W =  1231, *p* =  0.968), suggesting that just 2% of anglers are likely using treble hooks on the pier. The number of terminal tackle cited by anglers was predicted by assignment to the treatment group (β =  0.884, *p* =  0.004), as well as if they were fishing for food (β =  0.809, *p* =  0.007) (S3 Table). No significant predictors were identified for the number of terminal tackle cited by anglers, though fishing for food is significant at α =  0.10 (β =  0.434, *p* =  0.082).

### Pelican observations

A total of 157 anglers provided estimates of the number of pelicans they had seen in their section during their time on the pier. Only six anglers (4%) said they observed someone feeding pelicans on or near the pier, usually dead fish or used bait. Seventy-four anglers (47%) had been on the pier during the same period as pelican surveys were conducted, allowing for their subjective estimates to be more reliably compared with the objective pelican data conducted in their respective section. Of these anglers, 37 (50%) saw approximately the same number of pelicans that the pelican surveyors recorded in their respective sections. The majority of these were instances in which no pelicans were recorded and the anglers also reported seeing no pelicans in their area (S4 Table). Anglers whose estimates differed more substantially from the objective pelican data were more likely to overestimate (23 anglers; 31%) than underestimate (14 anglers; 19%) the number of pelicans in their area. The location of the angler on the pier did not appear to influence the accuracy of their pelican estimates (Fisher’s exact test, *p* =  0.165).

### Perceptions of entanglement and proposed solutions

**Fig 2:**
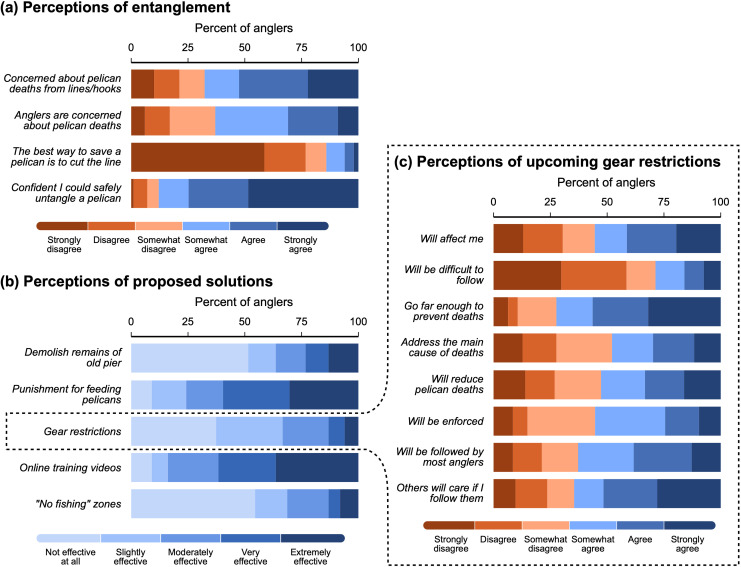
Perceptions of anglers as a whole. (a) Anglers’ agreement with statements about the pelican entanglement issue. (b) Anglers’ perceived effectiveness of various solutions that have been proposed to reduce pelican deaths on the pier. (c) Anglers’ agreement with statements about the upcoming fishing gear restrictions on the pier. Survey items/statements shortened for display purposes.

Overall, the majority of anglers are concerned about the entanglement issue, with 53% ‘agreeing’ or ‘strongly agreeing’ with the statement “I am concerned about the number of pelicans dying from fishing lines and hooks,” and just 21% ‘disagreeing’ or ‘strongly disagreeing’ with this statement (Fig 2a). Anglers are less sure if other anglers feel the same way, with only 31% ‘agreeing’ or ‘strongly agreeing’ that most anglers are concerned about the number of pelicans dying from fishing lines and hooks. However, 32% ‘somewhat agreed’ with this statement, indicating a slightly optimistic outlook on this descriptive norm amongst anglers. Anglers overwhelmingly disagree with the statement, “The best way to save a pelican caught in a fishing line is to cut the line as fast as possible,” with just 14% of anglers showing some level of agreement with this statement that contradicts best practice for saving a hooked pelican. Anglers also have a high degree of self-confidence in their abilities, with nearly 75% ‘agreeing’ or ‘strongly agreeing’ to the statement, “If a pelican was entangled in my fishing line, I am confident that I could safely untangle it.”

Online training videos have the greatest support from anglers amongst all proposed solutions to the pelican entanglement problem, with 62% expecting it to be ‘very’ or ‘extremely’ effective in reducing pelican deaths from entanglement (Fig 2b). Following closely behind the videos, greater enforcement or punishment for people caught feeding pelicans is the second-most effective solution according to anglers. “No fishing” zones and gear restrictions have the fewest number of anglers believing they would be ‘very’ or ‘extremely’ effective solutions (13%), though 49% of anglers admit that the gear restrictions could still be ‘slightly’ or ‘moderately’ effective at reducing pelican deaths. Seventy-three anglers stated that they were aware of the upcoming gear restrictions on the pier. Of the 23 anglers who were unaware of the restrictions, 17 (74%) enquired and were informed of the restrictions at the time of the survey, resulting in a total of 90 anglers who were permitted to answer the remaining questions regarding the gear restrictions. Overall, anglers were fairly split amongst their various opinions of the upcoming restrictions (Fig 2c). Despite 55% of anglers believing the restrictions would affect them, there was a slight tendency to hold more positive or optimistic perceptions of the restrictions. For example, most anglers at least ‘somewhat agreed’ that the new gear restrictions would reduce pelican deaths (53%) and be enforced (55%), and 71% disagreed to some extent that the restrictions would be difficult to follow. Anglers also anticipate relatively high compliance norms, with most agreeing to some extent that other anglers will follow the restrictions (descriptive norm; 63%) and that the people who are important to them will care if they follow the restrictions (subjective norm; 65%).

### Angler typologies

**Fig 3:**
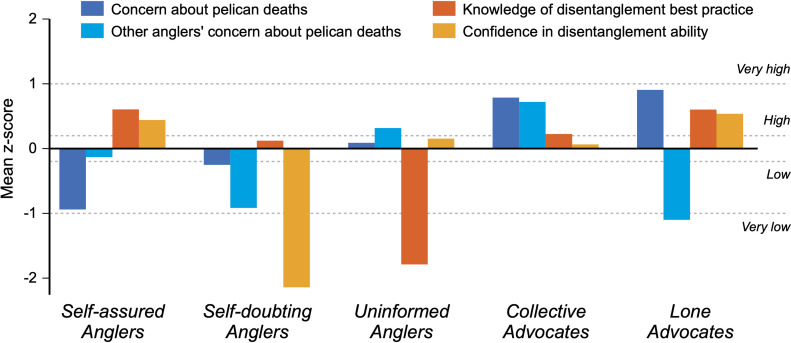
Classification of the five angler typologies on the South Skyway Fishing Pier. Mean standardized z-scores outside the 95% confidence interval of the mean (±0.20) are considered *high/low*, and scores beyond one standard deviation from the mean are considered *very high/low*. Survey items/statements shortened for display purposes.

Ninety-seven anglers answered all relevant questions for classification into typologies. These anglers were classified into the following five angler typologies (Fig 3):

- ***Self-assured anglers***: These anglers are unconcerned about the number of pelicans dying from fishing lines and hooks, but they know they shouldn’t cut the line if a pelican gets entangled in their line, and they are confident in their ability to safely untangle a pelican. These unconcerned but knowledgeable anglers represent 32% of participants.
- ***Self-doubting anglers***: These anglers also have a lesser concern about pelicans dying from fishing lines, and they believe most anglers are also unconcerned about the issue. Although their knowledge of best practices for saving a hooked bird is similar to the average angler, their confidence in their ability to safely untangle a pelican is extremely low. These unconcerned and pessimistic anglers represent 11% of participants.
- ***Uninformed anglers***: These anglers tend to believe other anglers care about pelicans dying from fishing lines, but they are the only typology characterized by a very poor knowledge of best practices when saving a hooked bird. These anglers represent a missed target of past messages, as they are far more likely to believe cutting the fishing line is the best thing to do when a pelican is hooked. They represent 19% of participants.
- ***Collective advocates***: These anglers are concerned about pelican deaths from fishing lines, and they believe most anglers share their concern. Their knowledge of best practices for saving a hooked bird is also slightly higher than the average angler. These concerned anglers that feel part of a larger community of concerned anglers represent 28% of participants.
- ***Lone advocates***: These anglers are concerned about pelican deaths, but unlike the *collective advocates*, they believe most anglers do not share their concern. Like the *self-assured anglers*, these anglers know that you should not cut the line if you hook a pelican and are confident in their ability to save a hooked bird. These concerned anglers that feel more unique within the angler community represent 10% of participants.

**Fig 4:**
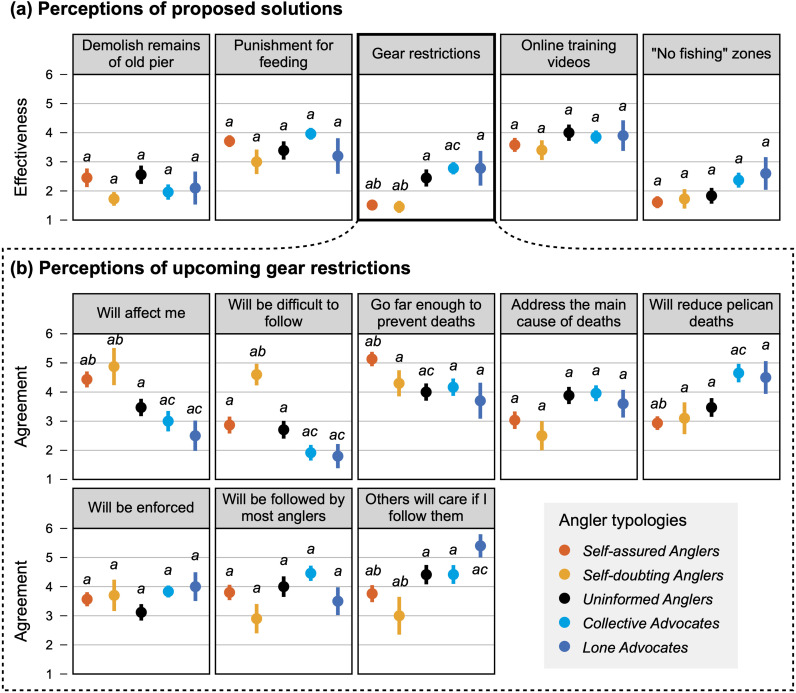
Comparison of psychosocial characteristics across angler typologies. (a) Mean effectiveness score for each of the five proposed solutions to entanglement-related pelican deaths in each typology. (b) Mean agreement to seven questions regarding the upcoming fishing gear restrictions in each typology. Error bars represent the standard error of the mean. Typology means with letters *b* and *c* are significantly different from each other at α =  0.05 (Dunn’s test). Survey items/statements shortened for display purposes.

No significant difference was found between different typologies’ perceptions of the effectiveness of the various proposed solutions to pelican entanglement, except for the gear restrictions (Fig 4a; S5 Table). Overall, *self-assured anglers* are frequently more similar to *self-doubting anglers* and most dissimilar to *collective advocates* and *lone advocates* when it comes to perceptions of the gear restrictions (Fig 4b; S6 Table). *Self-assured* and *self-doubting anglers* are more affected by these restrictions than both *collective* and *lone advocates*. When it comes to predictors of typology membership, *self-assured anglers* are more likely to view the gear restrictions as ineffective at reducing pelican deaths and believe that the current restrictions go far enough to protect pelicans (Table 5). These anglers are also more likely to fish alone than all other typologies. *Self-doubting anglers* also tend to think the effectiveness of the restrictions will be low, but they also believe the restrictions will be more difficult to follow. In stark contrast to these two types of anglers, the *collective advocates* are more likely to think the gear restrictions will be easy to follow and will reduce pelican deaths. These anglers are also the most likely to be going to the pier with friends or family members. The *lone advocates* do not have any additional statistically significant characteristics beyond what defines their typology, although with a larger sample size, their sense of a higher subjective compliance norm may emerge as a key characteristic (β =  0.725, *p* =  0.078). *Uninformed anglers* are most likely to view the gear restrictions as effective, though the model of membership into this typology has the poorest fit of all typologies (AIC =  87.62, McFadden *R*^*2*^ =  0.069). Notably, a Fisher’s exact test identified a significant difference among typologies in the frequency of anglers who fish for food (*p* =  0.038), yet this variable remained insignificant in all typology membership models. No other significant differences were identified between typologies regarding demographics, trip characteristics, or accuracy of pelican estimates (S5 and S6 Table).

**Table 5:** Coefficients of the most parsimonious models for membership in each angler typology.

Variable	*Self-assured Anglers*	*Self-doubting Anglers*	*Uninformed Anglers*	*Collective Advocates*	*Lone Advocates*
Fishing with others	-1.54 (0.58)^a^	2.06 (1.30)	0.91 (0.67)	1.60 (0.72)^a^	-1.01 (0.78)
Effectiveness of gear restrictions	-1.03 (0.32)^a^	-1.77 (0.91)^a^	0.59 (0.29)^a^		
Gear restrictions...					
... will be difficult to follow		1.10 (0.39)^a^		-0.56 (0.24)^a^	-0.75 (0.47)
... go far enough	0.46 (0.21)^a^				
... will reduce pelican deaths			-0.32 (0.22)	0.72 (0.23)^a^	
... address main cause of pelican deaths		0.31 (0.35)			
... others will care if I follow them					0.73 (0.41)^b^
AIC	89.47	39.44	87.62	82.99	51.77
McFadden pseudo *R*^*2*^	0.259	0.447	0.069	0.249	0.241

Coefficient estimates represented as *mean (SE)*.

^a^Significant at α =  0.05.

^b^Significant at α = 0.10.

## Discussion

The goals of this study were to provide a baseline understanding of the frequency of pelican entanglements at the South Skyway Fishing Pier, identify important factors that could contribute to higher risks of entanglement, and explore how differences amongst anglers could be used to craft targeted messages for behavior change. As expected, the gear restriction season (breeding season) had a much higher abundance of pelicans near the pier, and the frequency of injured or entangled pelicans was also higher—nearly double the frequency observed outside of the restriction season. The overall frequency of injured pelicans in this study (5.48%) is lower than previous estimates from 2019-2020 (8.70%) [14], which may be due, in part, to differences in survey frequency. Although there are typically fewer anglers on the pier during the restriction season, there is a noticeable relationship between anglers and pelicans. Both anglers and pelicans tend to favor the furthest end of the pier (sections A and B), but pelicans also appear to be drawn to areas with more anglers, regardless of their location. This may be indicative of a direct attraction to anglers, which presents some concern for entanglements. Entanglement risks will likely be highest when greater numbers of anglers are present, such as during mid-day and on weekends.

Risks may also be higher when many anglers are using multi-hook rigs (e.g., chicken or Sabiki rigs) and multi-hooks (e.g., double or treble hooks), though it is premature to estimate any type of relationship between gear use and frequency of entanglements. The results of this study suggest that roughly 75% of anglers may be using multi-hook rigs on the pier, and based on personal observations from the field, this may be a slight overestimate of the true share of anglers using this equipment. The estimated 2% of anglers using treble hooks, however, is almost certainly a large underestimate. Based on personal observations during angler surveying, treble hooks were common amongst anglers, particularly those using artificial lures. This underestimate is likely due to the small sample size of anglers [38] but may also reflect the need to include rarer items in the list of terminal tackle. Future studies would benefit from incorporating adapted versions of these gear questions into angler surveys to evaluate compliance rates during gear restriction seasons.

Based on the findings of angler’s perceptions, however, the outlook is not so grim. The vast majority of anglers know that cutting the line is an inappropriate solution when a pelican becomes entangled. This suggests the message, “Don’t cut the line,” from past campaigns has become salient to most anglers. Additionally, most anglers are at least somewhat concerned about pelican deaths from entanglement, and they appear to have a relatively accurate perception of pelican presence on the pier. This indicates that pelican entanglement is a relatively salient issue to anglers on the pier, and behavior change messages may not need to focus on building concern using emotional- or value-based messages (e.g., fear appraisals or altruistic frames), nor on challenging anglers’ perceptions of the extent of the problem (e.g., problem-severity frames) [31]. There is also evidence of relatively high descriptive norms of concern amongst anglers, as well as high descriptive and subjective norms of compliance with the gear restrictions. For most anglers, campaigns focused around activating these perceived emotional and behavioral ‘rules’ in the fishing community (e.g., social norm frames) may not be necessary. However, this analysis shows that all anglers are not the same, and the differences in opinions regarding gear restrictions highlight the polarizing nature of regulatory changes amongst anglers. These unique characteristics will require more targeted messages to increase regulatory compliance, improve their efficacy in rescuing a hooked pelican, and ultimately reduce the number of severe entanglements on the pier.

### Messaging for target audiences

Defining a target audience is a crucial preliminary step to any behavior change campaign. While it is frequently—and understandably—desirable to craft a single message for an entire population, some people may respond very differently to the same message, leading to ineffective or even unintentional outcomes [30]. Even within this narrow population of anglers in Tampa Bay, this study shows a high degree of variability between anglers that may influence how they respond to pelican entanglement messages. In this section, I offer recommendations for different messaging strategies that may be most effective amongst the different types of anglers identified in this analysis. These messages should be pilot tested, and their outcomes evaluated, in a subset of the angler population at the Skyway Pier to ensure they are achieving the desired outcomes. Critically, the behavior of interest in this context could be up for debate. Past campaigns have focused on how to safely untangle a hooked bird (direct entanglement focus), but other behaviors of interest could be avoiding illegal gear during the restriction season (compliance focus), or properly disposing of bait and/or tackle (indirect entanglement focus). It will be important to clearly define the target behavior for future campaigns, and this decision is best left to the stakeholders in this context. The recommendations outlined below are based upon a continuation of FWC’s desire to improve angler’s ability to rescue a hooked bird, but they could still be effectively applied to the other target behaviors that may be of interest.

**Fig 5:**
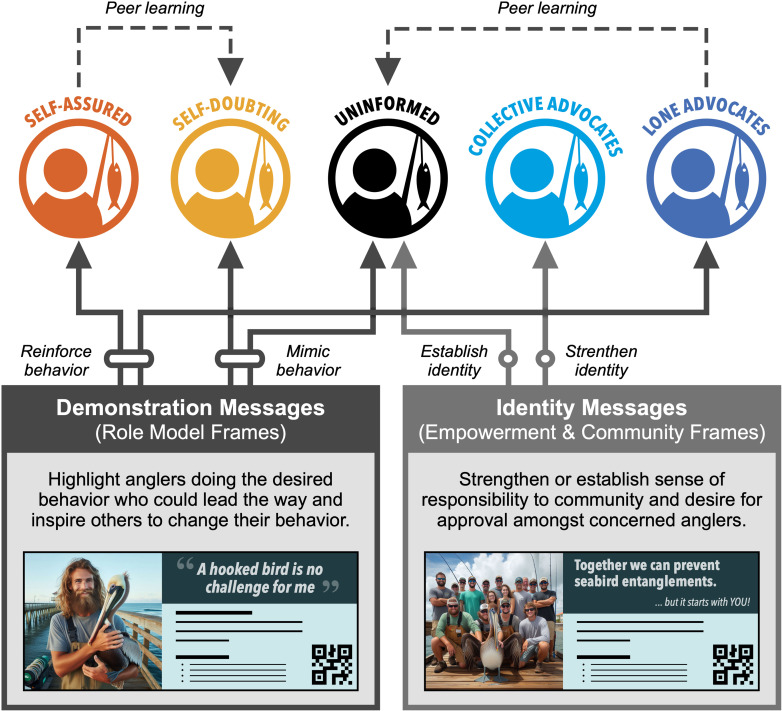
Recommended messages for target audiences on the Skyway Pier with examples. Arrows represent the anticipated path of direct (solid) and indirect (dashed) influence on different types of anglers. Photos created by Microsoft Bing Image Creator.

The first recommended messaging strategy is focused on harnessing the power and influence of potential champions of responsible fishing (Fig 5). Both *self-assured anglers* and *lone advocates* are highly knowledgeable of best practice in rescuing a hooked bird and confident in their ability to do so. Yet *self-assured anglers* remain unconcerned about the entanglement issue—potentially due to their high level of confidence in their own abilities—and *lone advocates* feel that they are one of the relative few that care about the issue. These types of anglers may be particularly responsive to messages that emphasize their responsibility to lead the way for others on the pier. Role model message frames, frequently used in demonstration messages, highlight individuals performing a desirable behavior and inspire others to do the same. Based on social learning theory [52], studies have shown that increasing the visibility of these role models can increase adoption of the behavior in others [53]. This approach could have multiple benefits for both the ‘teachers’ and the ‘learners’ in the population. These messages may encourage *self-assured anglers* and *lone advocates* to perform (or continue performing) the desired behavior, while encouraging them to increase this behavior in other anglers who may be less knowledgeable or less confident in their abilities. These messages can also provide direct behavioral benefits to the *self-doubting* and *uninformed anglers* by triggering conscious or subconscious behavioral mimicry [53,54]. In practice, this could take two different forms. Photos and quotes from influential anglers on the pier could present appeals to either the *self-doubting anglers* (using a *self-assured* role model) or the *uninformed anglers* (using a *lone advocate* role model) to learn how to safely unhook a seabird and spread the word to keep fishing alive and well on the pier.

A second messaging strategy is focused on building or re-orienting anglers’ identity (Fig 5). *Collective advocates* are one of the most unique types of anglers on the pier, as they are highly concerned about pelican deaths and believe that most others are, as well. For this target audience, messages based on empowerment and community frames may be most effective for promoting seabird-friendly behaviors. Empowerment frames emphasize one’s sense of agency and controllability [55,56], which could help counteract the potential risk of complacency in this audience. Community frames place this sense of self into a broader context by focusing on the impact of one’s actions on the well-being of the larger community [57]. In contrast to the *lone advocate*, the *collective advocate* likely feels part of the larger community of anglers, and these message frames could be effective in promoting the desired behavior in this audience by instilling a sense of responsibility to this community and their inherent desire to continue to be viewed favorably by people in that community. For example, a message targeting *collective advocates* could say, “Together we can prevent seabird entanglements. But it starts with *you*. Join your fellow anglers and learn what to do if you hook a bird.” Messages focusing on re-orienting one’s identity could also provide some benefit to *uninformed anglers* using social or collective identity frames [58,59]. These anglers have the least knowledge of how to rescue a hooked bird, yet they believe most anglers care about this issue. An identity-based message for this audience could take advantage of their potential desire to feel included in the ‘majority’ by connecting them to this ‘responsible angler’ identity and highlighting angler’s disapproval of those who do not know how to safely untangle a hooked bird. This could look something like, “62% of Skyway anglers agree: preventing pelican deaths is up to *you*. Do you know what to do? Don’t be left out. Find out now and join our community of responsible anglers.”

The strategies described above are broadly reflective of norm-based message frames, which aim to establish a sense of what is considered ‘acceptable’ behavior within a particular population [60]. Social norms are frequently recognized as one of the most powerful frames for promoting behavior change [61], but their use should not be taken lightly. The effects of norm-based messages can be highly variable depending upon what norm is activated and the recipient’s perception of the population for which that norm applies [30]. For most Skyway anglers, there is already a perceived norm of concern and regulatory compliance, so a more nuanced approach is likely needed to reach these audiences. Viewing these norm-based frames through more explicit lenses of leadership, responsibility, and identity, as described above, would more directly harness anglers’ overall sense of accountability and self-efficacy, as demonstrated by their overwhelming support of online training videos and greater enforcement or punishment for rule-breakers on the pier.

Importantly, FWC should not abandon current tactics, like the online education course and “Don’t cut the line” messaging. The results of this study suggest that anglers are supportive of efforts to educate and train anglers, and prior outreach campaigns have likely been effective in, at least, teaching anglers that they should not cut the line if they hook a bird. However, this message appears to have been missed by *uninformed anglers*, who make up almost one in five anglers on the pier. While this knowledge, alone, is likely insufficient to significantly reduce severe pelican entanglements, it may be an important stepping stone to increasing anglers’ ability to safely untangle a hooked bird. Education and training are especially important for both *uninformed* and *self-doubting anglers*. Future social marketing campaigns on the pier should use strategic messages to spur a particular call-to-action. All of the messages described in this section can be used alongside resources designed to increase anglers’ disentanglement ability and their confidence in their abilities, such as simple step-by-step instructions or a link/QR code to a video showing how to easily unhook a pelican. Calls-to-action with a digital fingerprint may be especially useful for tracking engagement with these messages, which could be valuable for evaluating outcomes of future behavior change campaigns.

### Limitations and future directions

This study serves as an important foundation for ongoing and future research into the extent of pelican entanglement and the potential effectiveness of gear restrictions at the Skyway Fishing Pier State Park. While these results provide a crucial baseline for both the extent of entanglement on the pier and angler’s perceptions of the issue, more research is needed to answer several outstanding questions. First, additional years of pelican data will be required to robustly quantify and compare trends in entanglement between regulatory seasons. Although prior data collected by Thomas and Forys [14] yield some insights into the extent of entanglement prior to the adoption of the new gear restrictions, the limited sample size makes it difficult to reliably establish baseline rates of pelican entanglement, injury, and death at the pier. Counterfactual thinking and causal inference techniques will be critical for estimating the effectiveness of the gear restrictions [62], and future impact evaluation studies will require both pelican entanglement data and estimates of angler’s use of regulated fishing gear. This study has showcased how both of these data can be collected, and future studies should adopt similar approaches with larger sample sizes.

Second, more data is needed to monitor how anglers’ perceptions may change over time. The results in this study are only reflective of anglers’ perceptions before the new gear restrictions were adopted on the pier. It is possible that anglers who were concerned about the restrictions, or who felt that they would be difficult to follow, may not have these same perceptions after the first year of restrictions. Additionally, it remains unclear if anglers’ perceptions of pelican abundance are a true reflection of their accuracy. Most anglers accurately estimated zero pelicans in areas where no pelicans were recorded. However, without asking these same questions during the breeding season, where true zeroes are much rarer, we cannot know for certain if anglers are accurately perceiving the number of pelicans near the pier or if there is a hidden underestimate bias. Tracking if, how, and for whom, opinions may change over time amongst anglers will be prudent for outreach efforts that can maximize angler’s evolving knowledge, compliance levels, and adoption of best practices for seabird-friendly fishing on the pier. This study was originally designed to collect data from anglers before, during, and after the gear restriction season over two years (N =  600). Unfortunately, permits to continue the angler surveys after the first pre-restriction period and to start the surveys again in September 2024 were denied without explanation. Future research efforts on the pier may also face similar obstacles, as social science continues to be underutilized in conservation and misunderstood by institutions globally [63]. Hopefully, the results discussed in this study will help break down these barriers and increase support for more research into the human dimensions of conservation and restoration, which will be crucial for solving modern environmental challenges.

## Supporting information

S1 File Angler survey (treatment and control versions). (PDF)

S1 Table Average number of pelicans counted per survey by season, time of day, day of the week, and section of the pier. Values expressed as *mean (SD)*.
(DOCX)

S2 Table Angler pressures by season, time of day, day of the week, and section of the pier. Values expressed as *mean (SD)*.
(DOCX)

S3 Table Results of the linear models predicting the number of tackle and terminal tackle used by anglers on the pier. (DOCX)

S4 Table Frequency of alignment between the number of pelicans recorded (objective) and the number of pelicans reported by anglers (subjective) in the same section of the pier. (DOCX)

S5 Table Results of the Kruskal-Wallis and Fisher’s exact tests on angler typologies. (DOCX)

S6 Table Results of Dunn’s test for pairwise typology comparisons of variables identified in the Kruskal-Wallis tests. (DOCX)
