# Supplementary material for: Angler perceptions of pelican entanglement reveal opportunities for seabird conservation on fishing piers in Tampa Bay
Source: PLoS One. 2025 Mar 25;20(3):e0320424. doi: 10.1371/journal.pone.0320424 (PMC11936238; doi:10.1371/journal.pone.0320424)
Supplement: S1 File — (PDF) [file pone.0320424.s001.pdf]

Q1. Have you completed this questionnaire before? (*Either online or in person*).

○ No *(go to Q2)* ○ Yes *(go to Q1a)*

Q1a. When was the last time you completed this questionnaire?

- ○ Earlier today *(end questionnaire)*
- ○ A week or two ago
- ○ About a month ago
- ○ Several months ago

First, we'd like to know who visits the South Skyway Fishing Pier. Please answer the questions below to tell us a bit about yourself.

Q2. What is your age? ____________________

Q3. Which of the following best describes your current employment status? *Select only one.*

- ○ Full-time
- ○ Part-time
- ○ Retired
- ○ Student
- ○ Unemployed

Q4. What is your gender?

- ○ Male
- ○ Female
- ○ Other

Q5. In the last 12 months, what was your total personal income before tax?

- ○ $25,000 or less
- ○ $25,001 - $50,000
- ○ $50,001 - $75,000
- ○ $75,001 - $100,000
- ○ $100,001 or more

Q6. Are you a Florida resident?

○ No *(go to Q6a)* ○ Yes *(go to Q7)*

Q6a. What is your primary reason for visiting Florida? *Select only one.*

- ○ Vacation or holiday
- ○ Visiting friends or family
- ○ Business or work-related activities
- ○ Something else

____

Q6b. What US state or country are you coming from?

____

(go to Q8)

Q7. What is the ZIP code of your primary residence?

____

Continue to next column →

Next, we'd like to know about your trip to the pier. The next few questions will help us understand what visitors like to do, how often they visit, and the different tackle they like to use.

Q8. What time of day have you been on the pier today? *You can select more than one time period if necessary.*

- ○ Morning (7am – 11am)
- ○ Mid-day (11am – 3pm)
- ○ Evening (3pm – 7pm)
- ○ Late night or early morning (7pm – 7am)

Q9. What is your reason for going to the South Skyway Fishing Pier? *Select all that apply.*

- ○ Fishing for fun or sport
- ○ Fishing for my own food
- ○ Fishing for food to sell
- ○ Watching friends or family fish
- ○ Something else

____

Q10. On this trip, did you go to the pier with anyone else? *Select all that apply.*

- ○ No, just me
- ○ Yes, with a friend
- ○ Yes, with a parent or sibling
- ○ Yes, with my spouse or significant other
- ○ Yes, with my children
- ○ Yes, with someone else

____

Q11. In a typical year, how often would you say you go to the South Skyway Fishing Pier?

- ○ At least once a week
- ○ Once or twice a month
- ○ A few times per year
- ○ This is my first time at the pier

Below is a map of the South Skyway Fishing Pier separated into five sections.

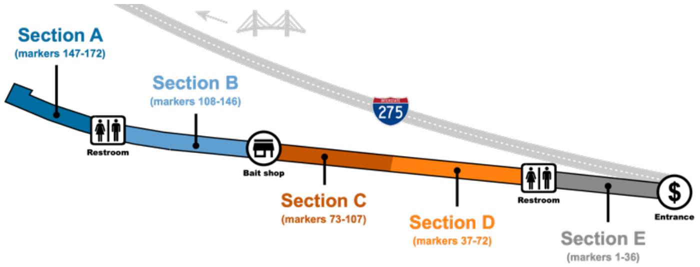

Q12. Based on the map, which section of the pier did you spend most of your time in? *If you aren't sure, give your best guess.*

- ○ **Section A** (markers 147-172)
- ○ **Section B** (markers 108-146)
- ○ **Section C** (markers 73-107)
- ○ **Section D** (markers 37-72)
- ○ **Section E** (markers 1-36)

Continue to next page →

CONTROL GROUP

Below are different types of tackle that some anglers use on the pier:

- Monofilament line
- Swivel
- Braided line
- Sinker or weight

Q13. Of these different types of tackle, how many did you use while you were at the South Skyway Fishing Pier?

- ◯ None of them
- ◯ One of them
- ◯ Two of them
- ◯ Three of them
- ◯ All of them

Below are different types of terminal tackle and bait that some anglers use on the pier:

- Bobber
- Circle hook
- Artificial bait (lures)
- Natural bait (live or dead)

Q14. Of these different types of terminal tackle and bait, how many did you use while you were at the South Skyway Fishing Pier?

- ◯ None of them
- ◯ One of them
- ◯ Two of them
- ◯ Three of them
- ◯ All of them

Continue to next column →

As you might know, some people are concerned about the number of pelicans that are injured or killed after getting entangled in fishing lines at the pier. We would like your help with collecting data on pelicans at the pier.

Q15. During this trip, did you see any pelicans on or near the pier?

◯ No *(go to Q16)* ◯ Yes *(go to Q15a)*

Q15a. If you had to guess, about how many pelicans did you see?

____

Q16. During this trip, did you see any pelicans that appeared to be injured or entangled by fishing lines?

◯ No *(go to Q17)* ◯ Yes *(go to Q16a)*

Q16a. If you had to guess, about how many injured or entangled pelicans did you see?

____

Q17. During this trip, did you see any dead pelicans near the pier?

◯ No *(go to Q18)* ◯ Yes *(go to Q17a)*

Q17a. If you had to guess, about how many dead pelicans did you see?

____

Q18. During this trip, did you see anyone feeding pelicans on or near the pier?

◯ No *(go to Q19)* ◯ Yes *(go to Q18a)*

Q18a. What was fed to the pelicans you saw? *Select all that apply.*

- ◯ Dead fish or used bait
- ◯ Chum
- ◯ Human food (bread, chips, popcorn, etc.)
- ◯ Something else ______________________________
- ◯ I'm not sure

Continue below ↓

Nearly done! We'd like to hear your opinions about pelican entanglement and some of the actions being taken to reduce pelican deaths at the South Skyway Fishing Pier. Please complete the remaining questions to let your voice be heard on this topic.

Q19. Please indicate the extent to which you agree or disagree with the statements below:

	Strongly disagree	*Disagree*	*Somewhat disagree*	*Somewhat agree*	*Agree*	*Strongly agree*
I am concerned about the number of pelicans dying from fishing lines and hooks.	◯	◯	◯	◯	◯	◯
If a pelican was entangled in my fishing line, I am confident that I could safely untangle it.	◯	◯	◯	◯	◯	◯
The best way to save a pelican caught in a fishing line is to cut the line as fast as possible.	◯	◯	◯	◯	◯	◯
Most anglers are concerned about the number of pelicans dying from fishing lines and hooks.	◯	◯	◯	◯	◯	◯

Continue to next page →

CONTROL GROUP

Below are some actions that could be taken to reduce pelican deaths from entanglement with fishing lines at the South Skyway Pier.

Q20. For each action, indicate how effective you think it would be at reducing pelican deaths from entanglement:

	Not effective at all	*Slightly effective*	*Moderately effective*	*Very effective*	*Extremely effective*
Limiting the gear that anglers can use on the pier.	○	○	○	○	○
Having a "no fishing zone" in parts of the pier with more pelicans.	○	○	○	○	○
Greater enforcement or punishment for people caught feeding pelicans on or near the pier.	○	○	○	○	○
Demolishing the remains of the old pier where many birds perch.	○	○	○	○	○
Create online videos showing how to safely untangle pelicans from fishing line.	○	○	○	○	○

For our last set of questions, we'd like to hear your opinions on the new gear restrictions approved by the Florida Fish and Wildlife Conservation Commission (FWC) that will go into effect this winter.

Q21. Are you aware of the upcoming new gear restrictions on the South Skyway Fishing Pier?

○ No *(end questionnaire)* ○ Yes *(go to Q22)*

Q22. Please indicate the extent to which you agree or disagree with the statements below:

	Strongly disagree	*Disagree*	*Somewhat disagree*	*Somewhat agree*	*Agree*	*Strongly agree*
The new restrictions will not affect me.	○	○	○	○	○	○
It will be difficult for me to follow the new restrictions.	○	○	○	○	○	○
The new restrictions do not go far enough to prevent pelican deaths on the pier.	○	○	○	○	○	○
The new restrictions do not address the main cause of pelican deaths.	○	○	○	○	○	○
The people who are important to me will care if I follow the new restrictions.	○	○	○	○	○	○
The new restrictions will probably reduce the number of pelican deaths from fishing lines.	○	○	○	○	○	○
There will be very little enforcement of the new restrictions.	○	○	○	○	○	○
Most anglers will follow the new restrictions.	○	○	○	○	○	○

Thank you for completing this questionnaire! Next time you visit the South Skyway Fishing Pier, please feel free to complete this survey again and contribute more data to our research efforts.

CONTROL GROUP

Q1. Have you completed this questionnaire before? (*Either online or in person*).

- ◯ No *(go to Q2)*
- ◯ Yes *(go to Q1a)*

Q1a. When was the last time you completed this questionnaire?

- ◯ Earlier today *(end questionnaire)*
- ◯ A week or two ago
- ◯ About a month ago
- ◯ Several months ago

First, we'd like to know who visits the South Skyway Fishing Pier. Please answer the questions below to tell us a bit about yourself.

Q2. What is your age? ______________________________

Q3. Which of the following best describes your current employment status? *Select only one.*

- ◯ Full-time
- ◯ Part-time
- ◯ Retired
- ◯ Student
- ◯ Unemployed

Q4. What is your gender?

- ◯ Male
- ◯ Female
- ◯ Other

Q5. In the last 12 months, what was your total personal income before tax?

- ◯ $25,000 or less
- ◯ $25,001 - $50,000
- ◯ $50,001 - $75,000
- ◯ $75,001 - $100,000
- ◯ $100,001 or more

Q6. Are you a Florida resident?

- ◯ No *(go to Q6a)*
- ◯ Yes *(go to Q7)*

Q6a. What is your primary reason for visiting Florida? *Select only one.*

- ◯ Vacation or holiday
- ◯ Visiting friends or family
- ◯ Business or work-related activities
- ◯ Something else ______________________________

Q6b. What US state or country are you coming from?

____

(go to Q8)

Q7. What is the ZIP code of your primary residence?

____

Continue to next column →

Next, we'd like to know about your trip to the pier. The next few questions will help us understand what visitors like to do, how often they visit, and the different tackle they like to use.

Q8. What time of day have you been on the pier today? *You can select more than one time period if necessary.*

- ◯ Morning (7am – 11am)
- ◯ Mid-day (11am – 3pm)
- ◯ Evening (3pm – 7pm)
- ◯ Late night or early morning (7pm – 7am)

Q9. What is your reason for going to the South Skyway Fishing Pier? *Select all that apply.*

- ◯ Fishing for fun or sport
- ◯ Fishing for my own food
- ◯ Fishing for food to sell
- ◯ Watching friends or family fish
- ◯ Something else ______________________________

Q10. On this trip, did you go to the pier with anyone else? *Select all that apply.*

- ◯ No, just me
- ◯ Yes, with a friend
- ◯ Yes, with a parent or sibling
- ◯ Yes, with my spouse or significant other
- ◯ Yes, with my children
- ◯ Yes, with someone else ______________________________

Q11. In a typical year, how often would you say you go to the South Skyway Fishing Pier?

- ◯ At least once a week
- ◯ Once or twice a month
- ◯ A few times per year
- ◯ This is my first time at the pier

Below is a map of the South Skyway Fishing Pier separated into five sections.

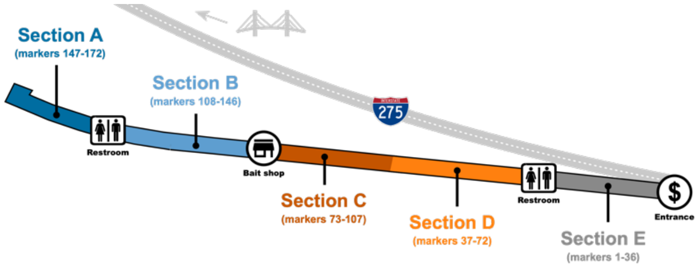

Q12. Based on the map, which section of the pier did you spend most of your time in? *If you aren't sure, give your best guess.*

- ◯ **Section A** (markers 147-172)
- ◯ **Section B** (markers 108-146)
- ◯ **Section C** (markers 73-107)
- ◯ **Section D** (markers 37-72)
- ◯ **Section E** (markers 1-36)

Continue to next page →

TREATMENT GROUP

TREATMENT GROUP

Below are different types of tackle that some anglers use on the pier:

- Monofilament line
- Swivel
- Braided line
- Sinker or weight
- Multi-hook rig (chicken rig, Sabiki rig, etc.)

Q13. Of these different types of tackle, how many did you use while you were at the South Skyway Fishing Pier?

○ None of them
○ One of them
○ Two of them
○ Three of them
○ Four of them
○ All of them

Below are different types of terminal tackle and bait that some anglers use on the pier:

- Bobber
- Circle hook
- Treble hook
- Artificial bait (lures)
- Natural bait (live or dead)

Q14. Of these different types of terminal tackle and bait, how many did you use while you were at the South Skyway Fishing Pier?

○ None of them
○ One of them
○ Two of them
○ Three of them
○ Four of them
○ All of them

Continue to next column →

As you might know, some people are concerned about the number of pelicans that are injured or killed after getting entangled in fishing lines at the pier. We would like your help with collecting data on pelicans at the pier.

Q15. During this trip, did you see any pelicans on or near the pier?

○ No *(go to Q16)*　○ Yes *(go to Q15a)*

Q15a. If you had to guess, about how many pelicans did you see?

____

Q16. During this trip, did you see any pelicans that appeared to be injured or entangled by fishing lines?

○ No *(go to Q17)*　○ Yes *(go to Q16a)*

Q16a. If you had to guess, about how many injured or entangled pelicans did you see?

____

Q17. During this trip, did you see any dead pelicans near the pier?

○ No *(go to Q18)*　○ Yes *(go to Q17a)*

Q17a. If you had to guess, about how many dead pelicans did you see?

____

Q18. During this trip, did you see anyone feeding pelicans on or near the pier?

○ No *(go to Q19)*　○ Yes *(go to Q18a)*

Q18a. What was fed to the pelicans you saw? *Select all that apply.*

○ Dead fish or used bait
○ Chum
○ Human food (bread, chips, popcorn, etc.)
○ Something else

____

○ I'm not sure

Continue below ↓

Nearly done! We'd like to hear your opinions about pelican entanglement and some of the actions being taken to reduce pelican deaths at the South Skyway Fishing Pier. Please complete the remaining questions to let your voice be heard on this topic.

Q19. Please indicate the extent to which you agree or disagree with the statements below:

	Strongly disagree	*Disagree*	*Somewhat disagree*	*Somewhat agree*	*Agree*	*Strongly agree*
I am concerned about the number of pelicans dying from fishing lines and hooks.	○	○	○	○	○	○
If a pelican was entangled in my fishing line, I am confident that I could safely untangle it.	○	○	○	○	○	○
The best way to save a pelican caught in a fishing line is to cut the line as fast as possible.	○	○	○	○	○	○
Most anglers are concerned about the number of pelicans dying from fishing lines and hooks.	○	○	○	○	○	○

Continue to next page →

Below are some actions that could be taken to reduce pelican deaths from entanglement with fishing lines at the South Skyway Pier.

Q20. For each action, indicate how effective you think it would be at reducing pelican deaths from entanglement:

	Not effective at all	*Slightly effective*	*Moderately effective*	*Very effective*	*Extremely effective*
Limiting the gear that anglers can use on the pier.	○	○	○	○	○
Having a "no fishing zone" in parts of the pier with more pelicans.	○	○	○	○	○
Greater enforcement or punishment for people caught feeding pelicans on or near the pier.	○	○	○	○	○
Demolishing the remains of the old pier where many birds perch.	○	○	○	○	○
Create online videos showing how to safely untangle pelicans from fishing line.	○	○	○	○	○

For our last set of questions, we'd like to hear your opinions on the new gear restrictions approved by the Florida Fish and Wildlife Conservation Commission (FWC) that will go into effect this winter.

Q21. Are you aware of the upcoming gear restrictions on the South Skyway Fishing Pier?

○ No *(end questionnaire)* ○ Yes *(go to Q22)*

Q22. Please indicate the extent to which you agree or disagree with the statements below:

	Strongly disagree	*Disagree*	*Somewhat disagree*	*Somewhat agree*	*Agree*	*Strongly agree*
The new restrictions will not affect me.	○	○	○	○	○	○
It will be difficult for me to follow the new restrictions.	○	○	○	○	○	○
The new restrictions do not go far enough to prevent pelican deaths on the pier.	○	○	○	○	○	○
The new restrictions do not address the main cause of pelican deaths.	○	○	○	○	○	○
The people who are important to me will care if I follow the new restrictions.	○	○	○	○	○	○
The new restrictions will probably reduce the number of pelican deaths from fishing lines.	○	○	○	○	○	○
There will be very little enforcement of the new restrictions.	○	○	○	○	○	○
Most anglers will follow the new restrictions.	○	○	○	○	○	○

Thank you for completing this questionnaire! Next time you visit the South Skyway Fishing Pier, please feel free to complete this survey again and contribute more data to our research efforts.

TREATMENT GROUP
