# Supplementary material for: Angler perceptions of pelican entanglement reveal opportunities for seabird conservation on fishing piers in Tampa Bay
Source: PLoS One. 2025 Mar 25;20(3):e0320424. doi: 10.1371/journal.pone.0320424 (PMC11936238; doi:10.1371/journal.pone.0320424)
Supplement: S1 Table — Values expressed as mean (SD). (DOCX) [file pone.0320424.s002.docx]

**S1 Table. Average number of pelicans counted per survey by season, time of day, day of the week, and section of the pier.**

| **Variable** | **Sample size** | **All pelicans** | | |  | **Adult pelicans** | | |  | **Juvenile pelicans** | | |
| --- | --- | --- | --- | --- | --- | --- | --- | --- | --- | --- | --- | --- |
|  |  | Total | Injured | Dead |  | Total | Injured | Dead |  | Total | Injured | Dead |
| Season |  |  |  |  |  |  |  |  |  |  |  |  |
| Restrictions | 35 | 45.00 (25.28) | 3.11 (2.69) | 0.06 (0.24) |  | 10.46 (8.61) | 0.63 (1.14) | 0.00 (0.00) |  | 34.54 (22.74) | 2.49 (2.54) | 0.06 (0.24) |
| Unrestricted | 34 | 34.18 (35.90) | 1.21 (1.63) | 0.00 (0.00) |  | 7.35 (9.43) | 0.18 (0.39) | 0.00 (0.00) |  | 26.82 (27.32) | 1.03 (1.45) | 0.00 (0.00) |
| Time of day |  |  |  |  |  |  |  |  |  |  |  |  |
| Morning | 34 | 34.15 (28.05) | 2.00 (2.64) | 0.03 (0.17) |  | 6.35 (6.72) | 0.35 (0.60) | 0.00 (0.00) |  | 27.79 (25.1) | 1.65 (2.48) | 0.03 (0.17) |
| Mid-day | 35 | 45.03 (33.55) | 2.34 (2.20) | 0.03 (0.17) |  | 11.43 (10.41) | 0.46 (1.09) | 0.00 (0.00) |  | 33.60 (25.36) | 1.89 (1.88) | 0.03 (0.17) |
| Day of week |  |  |  |  |  |  |  |  |  |  |  |  |
| Tuesday | 12 | 47.33 (24.93) | 2.92 (2.02) | 0.00 (0.00) |  | 10.33 (8.32) | 0.42 (0.51) | 0.00 (0.00) |  | 37.00 (21.75) | 2.50 (1.88) | 0.00 (0.00) |
| Wednesday | 14 | 41.21 (38.61) | 2.00 (2.48) | 0.00 (0.00) |  | 11.36 (10.62) | 0.29 (0.61) | 0.00 (0.00) |  | 29.86 (31.95) | 1.71 (2.37) | 0.00 (0.00) |
| Thursday | 2 | 39.00 (32.53) | 1.00 (1.41) | 0.00 (0.00) |  | 8.50 (4.95) | 0.00 (0.00) | 0.00 (0.00) |  | 30.50 (27.58) | 1.00 (1.41) | 0.00 (0.00) |
| Friday | 14 | 41.36 (29.55) | 2.71 (2.87) | 0.07 (0.27) |  | 9.29 (8.79) | 0.64 (0.74) | 0.00 (0.00) |  | 32.07 (24.43) | 2.07 (2.37) | 0.07 (0.27) |
| Saturday | 13 | 39.69 (37.51) | 1.46 (1.94) | 0.00 (0.00) |  | 7.38 (11.29) | 0.62 (1.66) | 0.00 (0.00) |  | 32.31 (27.57) | 0.85 (1.34) | 0.00 (0.00) |
| Sunday | 14 | 29.93 (25.81) | 2.00 (2.72) | 0.07 (0.27) |  | 6.43 (6.86) | 0.14 (0.36) | 0.00 (0.00) |  | 23.5 (21.22) | 1.86 (2.71) | 0.07 (0.27) |
| Pier section |  |  |  |  |  |  |  |  |  |  |  |  |
| A | 69 | 15.93 (14.83) | 0.78 (1.11) | 0.00 (0.00) |  | 3.07 (3.97) | 0.13 (0.34) | 0.00 (0.00) |  | 12.86 (11.99) | 0.65 (1.00) | 0.00 (0.00) |
| B | 69 | 8.59 (10.53) | 0.59 (1.22) | 0.01 (0.12) |  | 1.86 (2.99) | 0.10 (0.39) | 0.00 (0.00) |  | 6.74 (8.87) | 0.49 (1.18) | 0.01 (0.12) |
| C | 69 | 7.00 (8.62) | 0.42 (0.83) | 0.01 (0.12) |  | 1.65 (2.87) | 0.13 (0.57) | 0.00 (0.00) |  | 5.35 (6.85) | 0.29 (0.62) | 0.01 (0.12) |
| D | 69 | 8.14 (7.67) | 0.38 (0.64) | 0.00 (0.00) |  | 2.35 (3.78) | 0.04 (0.21) | 0.00 (0.00) |  | 5.80 (5.30) | 0.33 (0.63) | 0.00 (0.00) |

Values expressed as *mean (SD)*.
