# Supplementary material for: Angler perceptions of pelican entanglement reveal opportunities for seabird conservation on fishing piers in Tampa Bay
Source: PLoS One. 2025 Mar 25;20(3):e0320424. doi: 10.1371/journal.pone.0320424 (PMC11936238; doi:10.1371/journal.pone.0320424)
Supplement: S2 Table — Values expressed as mean (SD). (DOCX) [file pone.0320424.s003.docx]

**S2 Table. Angler pressures by season, time of day, day of the week, and section of the pier.**

| **Variable** | **Sample size** | **Number of active anglers** | **Number of fishing lines in water** | **Lines per angler** |
| --- | --- | --- | --- | --- |
| Season |  |  |  |  |
| Restrictions | 35 | 42.71 (30.37) | 53.66 (37.02) | 1.29 (0.23) |
| Unrestricted | 34 | 93.35 (61.76) | 117.74 (73.78) | 1.29 (0.11) |
| Time of day |  |  |  |  |
| Morning | 34 | 58.24 (52.98) | 74.29 (65.31) | 1.30 (0.17) |
| Mid-day | 35 | 76.83 (55.00) | 95.86 (66.04) | 1.29 (0.19) |
| Day of week |  |  |  |  |
| Tuesday | 12 | 36.33 (21.28) | 49.25 (26.74) | 1.44 (0.27) |
| Wednesday | 14 | 36.21 (22.78) | 44.43 (24.22) | 1.29 (0.17) |
| Thursday | 2 | 94.00 (42.43) | 118.5 (38.89) | 1.30 (0.17) |
| Friday | 14 | 45.43 (20.51) | 57.14 (27.78) | 1.23 (0.14) |
| Saturday | 13 | 98.62 (44.82) | 124.23 (59.05) | 1.23 (0.12) |
| Sunday | 14 | 115.71 (78.27) | 144.00 (91.13) | 1.28 (0.12) |
| Pier section |  |  |  |  |
| A | 69 | 23.19 (16.92) | 29.71 (21.16) | 1.30 (0.34) |
| B | 69 | 21.90 (18.29) | 27.90 (22.58) | 1.33 (0.45) |
| C | 69 | 12.62 (11.73) | 15.54 (14.36) | 1.16 (0.44) |
| D | 69 | 9.96 (11.61) | 12.09 (13.16) | 1.25 (0.50) |

Values expressed as *mean (SD)*.
