# Supplementary material for: Angler perceptions of pelican entanglement reveal opportunities for seabird conservation on fishing piers in Tampa Bay
Source: PLoS One. 2025 Mar 25;20(3):e0320424. doi: 10.1371/journal.pone.0320424 (PMC11936238; doi:10.1371/journal.pone.0320424)
Supplement: S3 Table — (DOCX) [file pone.0320424.s004.docx]

**S3 Table. Results of the linear models predicting the number of tackle and terminal tackle used by anglers on the pier.**

| **Variable** | **Tackle gear** | |  | **Terminal tackle gear** | |
| --- | --- | --- | --- | --- | --- |
|  | Coefficient (SE) | *p*-value |  | Coefficient (SE) | *p*-value |
| (Intercept) | 1.85 (0.81) | 0.025 |  | 2.05 (0.69) | 0.004 |
| Treatment group | 0.88 (0.29) | 0.004 |  | 0.14 (0.25) | 0.579 |
| Age | 0.00 (0.01) | 0.945 |  | 0.00 (0.01) | 0.579 |
| Male | 0.42 (0.39) | 0.279 |  | 0.20 (0.33) | 0.536 |
| Fishing for food | 0.81 (0.29) | 0.007 |  | 0.43 (0.25) | 0.082 |
| Income |  |  |  |  |  |
| $25,000 or less | 0.20 (0.63) | 0.756 |  | 0.44 (0.54) | 0.413 |
| $25,001 - $50,000 | 0.18 (0.53) | 0.740 |  | 0.28 (0.45) | 0.538 |
| $50,001 - $75,000 | 0.34 (0.48) | 0.490 |  | -0.04 (0.41) | 0.925 |
| $75,001 - $100,000 | -0.30 (0.54) | 0.574 |  | 0.00 (0.46) | 0.999 |
| Sample size | 88 |  |  | 88 |  |
| Multiple *R^2^* | 0.213 |  |  | 0.061 |  |
