# Supplementary material for: Angler perceptions of pelican entanglement reveal opportunities for seabird conservation on fishing piers in Tampa Bay
Source: PLoS One. 2025 Mar 25;20(3):e0320424. doi: 10.1371/journal.pone.0320424 (PMC11936238; doi:10.1371/journal.pone.0320424)
Supplement: S4 Table — (DOCX) [file pone.0320424.s005.docx]

**S4 Table. Frequency of alignment between the number of pelicans recorded (objective) and the number of pelicans reported by anglers (subjective) in the same section of the pier.**

| **Objective number of pelicans** | **Subjective number of pelicans** | **Accuracy** | **Frequency** |
| --- | --- | --- | --- |
| 0 | 0 | About right | 39% |
| 0 | 1-4 | Overestimate | 8% |
| 0 | 5-9 | Overestimate | 3% |
| 0 | 10-19 | Overestimate | 5% |
| 0 | 20+ | Overestimate | 3% |
| 1-4 | 0 | Underestimate | 19% |
| 1-4 | 1-4 | About right | 11% |
| 1-4 | 5-9 | Overestimate | 1% |
| 1-4 | 10-19 | Overestimate | 3% |
| 1-4 | 20+ | Overestimate | 5% |
| 5-9 | 0 | Underestimate | 0% |
| 5-9 | 1-4 | Underestimate | 0% |
| 5-9 | 5-9 | About right | 0% |
| 5-9 | 10-19 | Overestimate | 0% |
| 5-9 | 20+ | Overestimate | 3% |
