# Supplementary material for: Angler perceptions of pelican entanglement reveal opportunities for seabird conservation on fishing piers in Tampa Bay
Source: PLoS One. 2025 Mar 25;20(3):e0320424. doi: 10.1371/journal.pone.0320424 (PMC11936238; doi:10.1371/journal.pone.0320424)
Supplement: S5 Table — (DOCX) [file pone.0320424.s006.docx]

**S5 Table. Results of the Kruskal-Wallis (continuous variables) and Fisher’s exact tests (categorical variables) on angler typologies.**

| **Variable** | **Kruskal-Wallis test^a^** | | **Fisher’s exact test** |
| --- | --- | --- | --- |
|  | χ^2^ | *p*-value | *p*-value |
| Proposed solutions |  |  |  |
| Gear restrictions | 24.45 | < 0.001 |  |
| "No fishing" zone | 8.25 | 0.083 |  |
| Punishment for feeding | 4.81 | 0.307 |  |
| Demolishing old pier | 2.98 | 0.561 |  |
| Online training videos | 3.41 | 0.491 |  |
| Gear restrictions... |  |  |  |
| ... will affect me | 19.29 | < 0.001 |  |
| ... will be difficult to follow | 24.54 | < 0.001 |  |
| ... go far enough | 13.93 | 0.008 |  |
| ... address main cause of deaths | 10.69 | 0.030 |  |
| ... will reduce pelican deaths | 19.07 | 0.001 |  |
| ... will be enforced | 3.58 | 0.466 |  |
| ... will be followed by anglers | 9.12 | 0.058 |  |
| ... others will care if I follow | 13.74 | 0.008 |  |
| Demographics |  |  |  |
| Age | 4.61 | 0.330 |  |
| Income |  |  | 0.219 |
| Trip to pier |  |  |  |
| Section of pier |  |  | 0.456 |
| Frequency of visits |  |  | 0.124 |
| Fishing for food |  |  | 0.038 |
| Fishing with others |  |  | 0.013 |
| Pelican estimate |  |  | 0.803 |

^a^ d.f. = 4
