# Supplementary material for: Angler perceptions of pelican entanglement reveal opportunities for seabird conservation on fishing piers in Tampa Bay
Source: PLoS One. 2025 Mar 25;20(3):e0320424. doi: 10.1371/journal.pone.0320424 (PMC11936238; doi:10.1371/journal.pone.0320424)
Supplement: S6 Table — (DOCX) [file pone.0320424.s007.docx]

**S6 Table. Results of Dunn’s test for pairwise typology comparisons of variables identified as significant in the Kruskal-Wallis tests.**

| **Variable** | **Typology comparison** | **Z-score** | **p-value** | **Adj. p-value** |
| --- | --- | --- | --- | --- |
| Effectiveness of gear restrictions | Self-assured—Self-doubting | 0.21 | 0.837 | 1 |
|  | Self-assured—Uninformed | -2.69 | 0.007 | 0.072 |
|  | Self-assured—Collective Advocate | -4.28 | < 0.001 | < 0.001 |
|  | Self-assured—Lone Advocate | -2.24 | 0.025 | 0.251 |
|  | Self-doubting—Uninformed | 2.27 | 0.023 | 0.232 |
|  | Self-doubting—Collective Advocate | 3.35 | 0.001 | 0.008 |
|  | Self-doubting—Lone Advocate | -2.05 | 0.041 | 0.407 |
|  | Uninformed—Collective Advocate | 1.08 | 0.28 | 1 |
|  | Uninformed—Lone Advocate | -0.13 | 0.9 | 1 |
|  | Collective Advocate—Lone Advocate | 0.72 | 0.471 | 1 |
| Gear restrictions will affect me | Self-assured—Self-doubting | -0.77 | 0.442 | 1 |
|  | Self-assured—Uninformed | 1.99 | 0.047 | 0.47 |
|  | Self-assured—Collective Advocate | 3.07 | 0.002 | 0.021 |
|  | Self-assured—Lone Advocate | 3.09 | 0.002 | 0.02 |
|  | Self-doubting—Uninformed | -2.12 | 0.034 | 0.34 |
|  | Self-doubting—Collective Advocate | -2.81 | 0.005 | 0.05 |
|  | Self-doubting—Lone Advocate | 3.02 | 0.003 | 0.025 |
|  | Uninformed—Collective Advocate | -0.75 | 0.453 | 1 |
|  | Uninformed—Lone Advocate | 1.32 | 0.188 | 1 |
|  | Collective Advocate—Lone Advocate | 0.76 | 0.447 | 1 |
| Gear restrictions will be difficult to follow | Self-assured—Self-doubting | -2.76 | 0.006 | 0.057 |
|  | Self-assured—Uninformed | 0 | 0.996 | 1 |
|  | Self-assured—Collective Advocate | 2.41 | 0.016 | 0.159 |
|  | Self-assured—Lone Advocate | 2.1 | 0.036 | 0.357 |
|  | Self-doubting—Uninformed | -2.53 | 0.011 | 0.113 |
|  | Self-doubting—Collective Advocate | -4.43 | < 0.001 | < 0.001 |
|  | Self-doubting—Lone Advocate | 3.97 | < 0.001 | 0.001 |
|  | Uninformed—Collective Advocate | -2.08 | 0.038 | 0.376 |
|  | Uninformed—Lone Advocate | 1.92 | 0.055 | 0.547 |
|  | Collective Advocate—Lone Advocate | 0.28 | 0.777 | 1 |
| Gear restrictions go far enough | Self-assured—Self-doubting | 1.83 | 0.067 | 0.669 |
|  | Self-assured—Uninformed | 3.05 | 0.002 | 0.023 |
|  | Self-assured—Collective Advocate | 2.72 | 0.007 | 0.066 |
|  | Self-assured—Lone Advocate | 2.53 | 0.011 | 0.114 |
|  | Self-doubting—Uninformed | -0.65 | 0.518 | 1 |
|  | Self-doubting—Collective Advocate | -0.2 | 0.842 | 1 |
|  | Self-doubting—Lone Advocate | 0.57 | 0.569 | 1 |
|  | Uninformed—Collective Advocate | 0.58 | 0.564 | 1 |
|  | Uninformed—Lone Advocate | -0.01 | 0.994 | 1 |
|  | Collective Advocate—Lone Advocate | 0.48 | 0.632 | 1 |
| Gear restrictions address main cause of deaths | Self-assured—Self-doubting | 0.97 | 0.331 | 1 |
|  | Self-assured—Uninformed | -1.9 | 0.057 | 0.571 |
|  | Self-assured—Collective Advocate | -2.25 | 0.025 | 0.246 |
|  | Self-assured—Lone Advocate | -1.04 | 0.3 | 1 |
|  | Self-doubting—Uninformed | 2.34 | 0.019 | 0.193 |
|  | Self-doubting—Collective Advocate | 2.58 | 0.01 | 0.099 |
|  | Self-doubting—Lone Advocate | -1.64 | 0.101 | 1 |
|  | Uninformed—Collective Advocate | 0.12 | 0.905 | 1 |
|  | Uninformed—Lone Advocate | 0.5 | 0.618 | 1 |
|  | Collective Advocate—Lone Advocate | 0.63 | 0.529 | 1 |
| Gear restrictions will reduce pelican deaths | Self-assured—Self-doubting | -0.34 | 0.736 | 1 |
|  | Self-assured—Uninformed | -1.04 | 0.296 | 1 |
|  | Self-assured—Collective Advocate | -3.87 | < 0.001 | 0.001 |
|  | Self-assured—Lone Advocate | -2.65 | 0.008 | 0.08 |
|  | Self-doubting—Uninformed | 0.49 | 0.626 | 1 |
|  | Self-doubting—Collective Advocate | 2.51 | 0.012 | 0.121 |
|  | Self-doubting—Lone Advocate | -1.89 | 0.059 | 0.587 |
|  | Uninformed—Collective Advocate | 2.36 | 0.018 | 0.181 |
|  | Uninformed—Lone Advocate | -1.63 | 0.102 | 1 |
|  | Collective Advocate—Lone Advocate | 0.28 | 0.782 | 1 |
| Gear restrictions will be followed by anglers | Self-assured—Self-doubting | 1.6 | 0.111 | 1 |
|  | Self-assured—Uninformed | -0.41 | 0.685 | 1 |
|  | Self-assured—Collective Advocate | -1.72 | 0.086 | 0.856 |
|  | Self-assured—Lone Advocate | 0.59 | 0.552 | 1 |
|  | Self-doubting—Uninformed | 1.77 | 0.077 | 0.767 |
|  | Self-doubting—Collective Advocate | 2.8 | 0.005 | 0.051 |
|  | Self-doubting—Lone Advocate | -0.82 | 0.414 | 1 |
|  | Uninformed—Collective Advocate | 1.1 | 0.272 | 1 |
|  | Uninformed—Lone Advocate | 0.85 | 0.393 | 1 |
|  | Collective Advocate—Lone Advocate | 1.83 | 0.068 | 0.676 |
| Others will care if I follow the gear restrictions | Self-assured—Self-doubting | 0.86 | 0.388 | 1 |
|  | Self-assured—Uninformed | -1.33 | 0.185 | 1 |
|  | Self-assured—Collective Advocate | -1.58 | 0.114 | 1 |
|  | Self-assured—Lone Advocate | -3.05 | 0.002 | 0.023 |
|  | Self-doubting—Uninformed | 1.81 | 0.07 | 0.7 |
|  | Self-doubting—Collective Advocate | 2 | 0.045 | 0.454 |
|  | Self-doubting—Lone Advocate | -3.21 | 0.001 | 0.013 |
|  | Uninformed—Collective Advocate | 0.1 | 0.922 | 1 |
|  | Uninformed—Lone Advocate | -1.79 | 0.073 | 0.73 |
|  | Collective Advocate—Lone Advocate | -1.82 | 0.069 | 0.694 |
